# Supplementary material for: Structural basis for gating mechanism of the human sodium-potassium pump
Source: Nat Commun. 2022 Sep 8;13:5293. doi: 10.1038/s41467-022-32990-x (PMC9458724; doi:10.1038/s41467-022-32990-x)
Supplement: Supplementary file 1 — Supplementary Information [file 41467_2022_32990_MOESM1_ESM.pdf]

Structural basis for gating mechanism of the human sodium-potassium pump

Phong T. Nguyen[1*], Christine Deisl[2], Michael Fine[2], Trevor S. Tippetts[3], Emiko Uchikawa[4], Xiao-chen Bai[4*], Beth Levine[1]

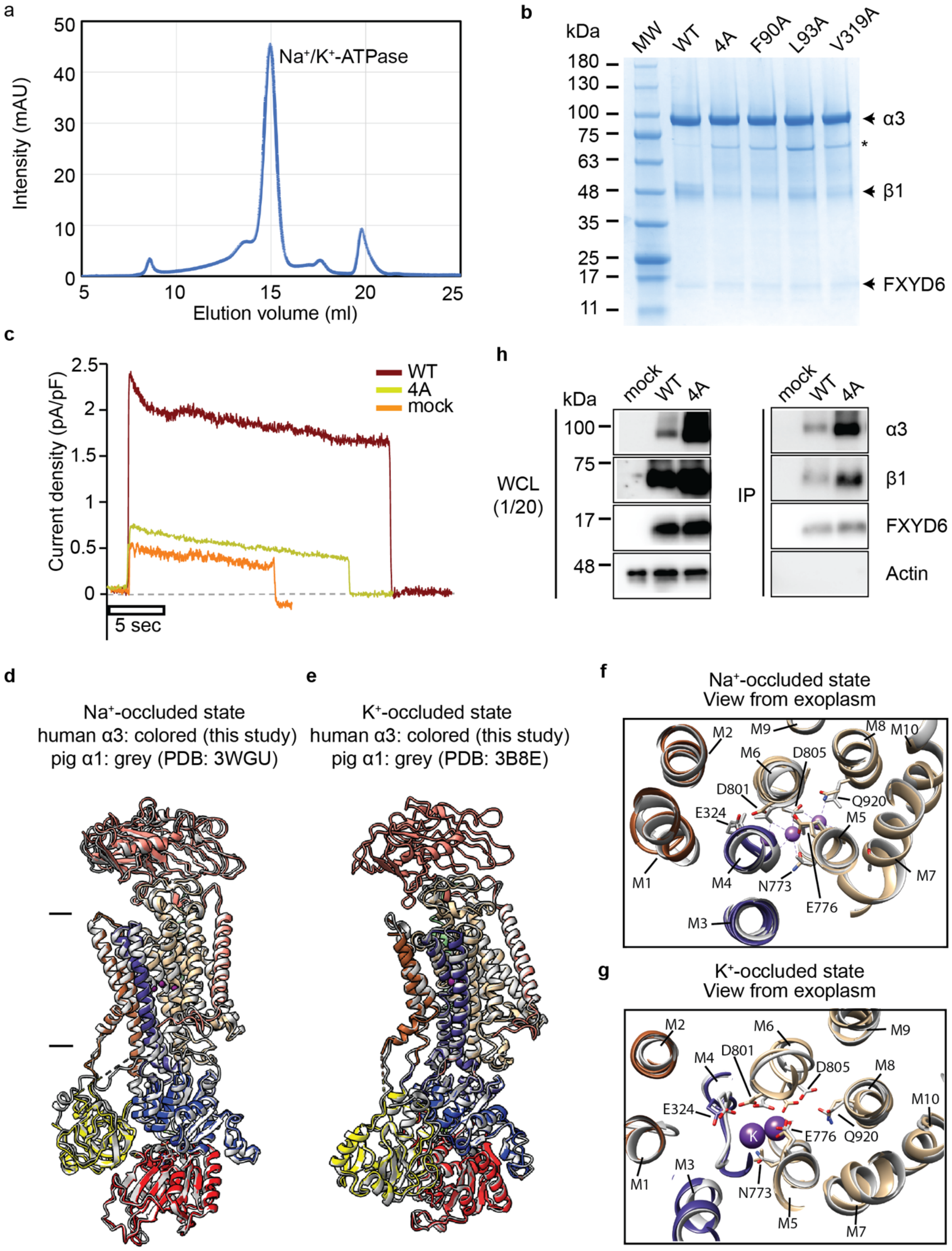

a
Na+/K+-ATPase
Intensity (mAU)
Elution volume (ml)
b
kDa
MW WT 4A F90A L93A V319A
α3
*
β1
FXYD6
c
WT
4A
mock
Current density (pA/pF)
5 sec
h
kDa
mock WT 4A
WCL (1/20)
IP
α3
β1
FXYD6
Actin
d
Na+-occluded state
human α3: colored (this study)
pig α1: grey (PDB: 3WGU)
e
K+-occluded state
human α3: colored (this study)
pig α1: grey (PDB: 3B8E)
f
Na+-occluded state
View from exoplasm
g
K+-occluded state
View from exoplasm

Supplementary Fig. 1: Functional and structural characterization of the human α3 Na^+/K^+-ATPase. (**a, b**) Size-exclusion chromatography **(a)** and SDS-PAGE **(b)** of the purified human α3 Na^+/K^+-ATPase. *: contaminant. Experiments were done in triplicate. (**c)** Whole-cell patch-clamp electrophysiological traces of the wild-type (WT) and cation-binding deficient (4A) mutant Na^+/K^+-ATPase**.** The dashed line indicates the zero-current x-axis. **(d, f**) Comparison of the overall structure (**d**) and cation-binding sites (**f**) of the human α3 (colored) (this study) and the pig α1 (grey) (PDB: 3WGU) structure in their Na^+-occluded state. The black dashed line represents missing density of amino acid residues 261 – 270 in the human Na^+-occluded state structural model. (**e**, **g**) Comparison of the overall structure (**e**) and cation-binding sites (**g**) of the human α3 (colored) (this study) and the pig α1 (grey) (PDB: 3B8E) structure in their K^+-occluded state. (**h**) Representative western-blot images of the cell surface protein expression of the wild-type and mutant Na^+/K^+-ATPase variants. WCL: whole-cell lysate. IP: immunoprecipitation. Experiments were done in triplicate.

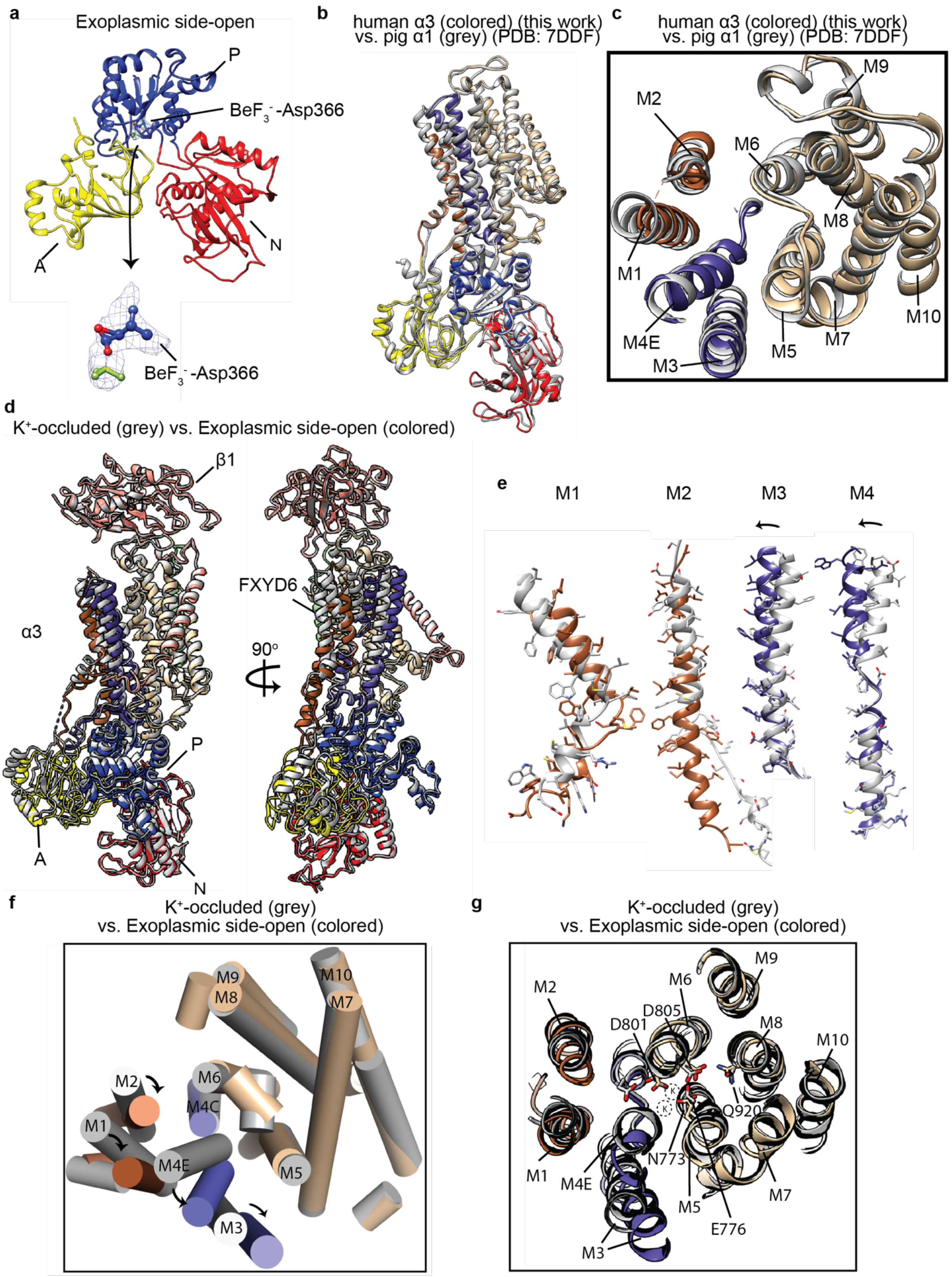
a
Exoplasmic side-open
P
BeF$_3^-$-Asp366
A
N
BeF$_3^-$-Asp366
b
human α3 (colored) (this work)
vs. pig α1 (grey) (PDB: 7DDF)
c
human α3 (colored) (this work)
vs. pig α1 (grey) (PDB: 7DDF)
M9
M2
M6
M8
M1
M10
M4E
M5
M7
M3
d
K$^+$-occluded (grey) vs. Exoplasmic side-open (colored)
β1
FXYD6
α3
90°
P
A
N
e
M1
M2
M3
M4
f
K$^+$-occluded (grey)
vs. Exoplasmic side-open (colored)
M9
M8
M10
M7
M2
M6
M4C
M1
M4E
M5
M3
g
K$^+$-occluded (grey)
vs. Exoplasmic side-open (colored)
M9
M2
M6
D805
M8
D801
M10
Q920
N773
M1
M4E
M5
M7
E776
M3

Supplementary Fig. 2: Structural features of the human α3 Na^+/K^+-ATPase in its exoplasmic side-open. **(a)** The cytoplasmic domains trapped by BeF_3^-. Mesh: electron density of BeF_3^-. **(b, c)** Comparison of the overall structure **(b)** and transmembrane helices **(c)** of the human α3 (this study) (colored) and the pig α1 (PDB: 7DDF) (grey) structure in their exoplasmic side-open state. **(d, e, f, g)** Superposition of the overall structure **(d)**, M1 – M4 helices (close-up) **(e)**, transmembrane helices **(f)** and cation-binding sites **(g)** of the human α3 K^+-occluded (this study) and exoplasmic side-open (this study). Dash circles labeled "K" in **(g)** indicated the bound K^+ ions in the K^+-occluded state. The N773, E776, D801, and D805 were modeled for the purpose of comparison to illustrate the side chain movement between states.

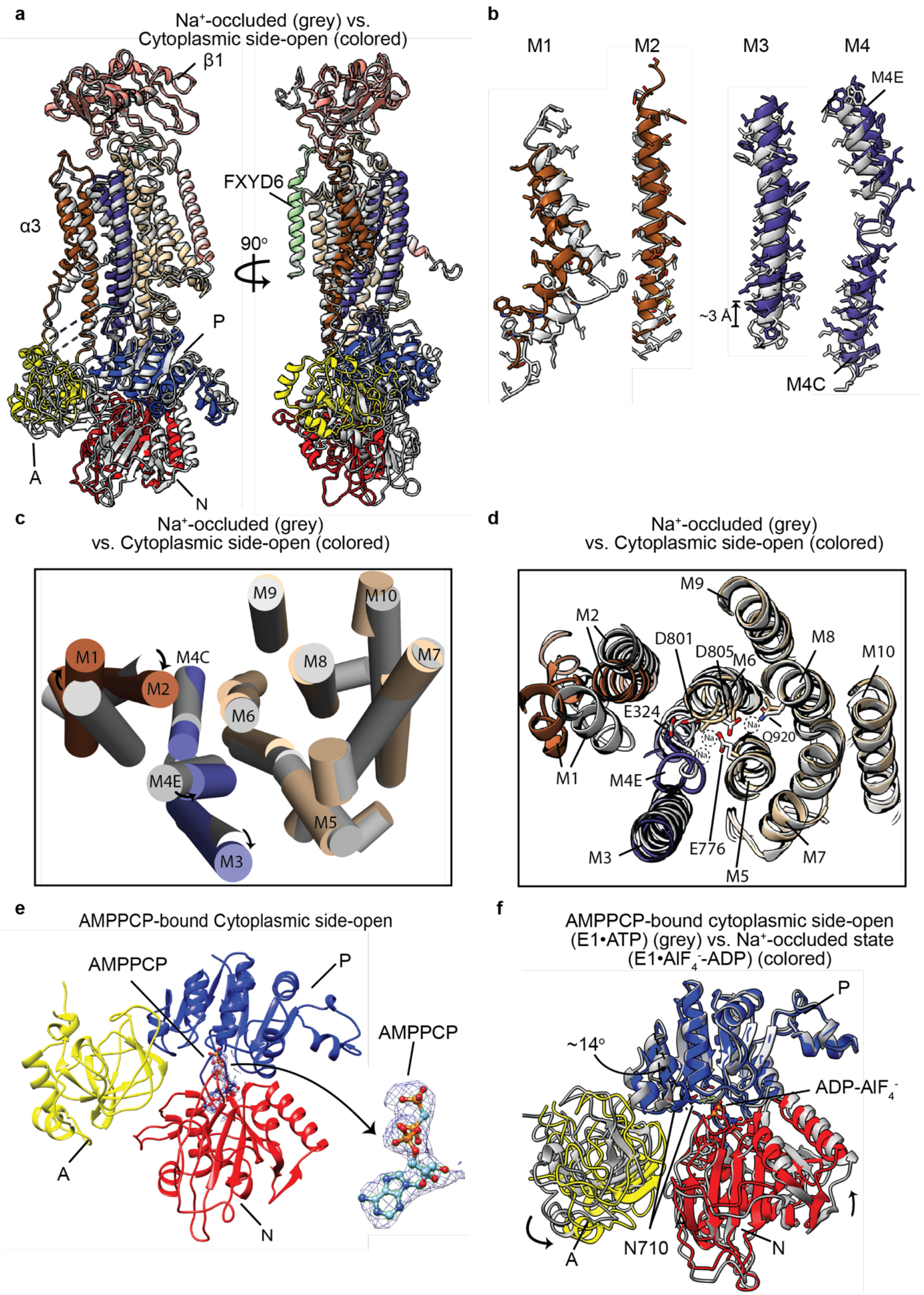

Supplementary Fig. 3: Conformational changes of the human α3 Na^+/K^+-ATPase among the

cytoplasmic side-open, AMPPCP-bound cytoplasmic side-open and Na^+-occluded states. (**a, b**) Superimpose the overall structures of the cytoplasmic side-open (colored) and Na^+-occluded (grey) structures by aligning their M7 – M10 helices. The black dashed lines represent missing density of amino acid residues 261 – 270 and 260 - 271 in the Na^+-occluded and the cytoplasmic side-open structural models, respectively. **(b)** A close-up view of the helices M1 - M4 from the superimposed overall structures in panel (a). **(c, d)** Superimpose the transmembrane helices (**c**) and the Na^+ binding sites (**d**) by aligning their M7 – M10 helices in the cytoplasmic side-open (colored) and Na^+-occluded (grey) states. Dash circles labeled "Na" indicated the bound Na^+ in the Na^+-occluded states. The N773, E776, D801, and D805 were modeled for the purpose of comparison to illustrate the side chain movement between states. **(e)** The cytoplasmic domains trapped by AMPPCP. Mesh: electron density of AMPPCP. **(f)** Superposition of the cytoplasmic domains of the AMPPCP-bound cytoplasmic side-open (E1•ATP) (grey) and Na^+-occluded state (E1•P-ADP) (colored) by the P domain.

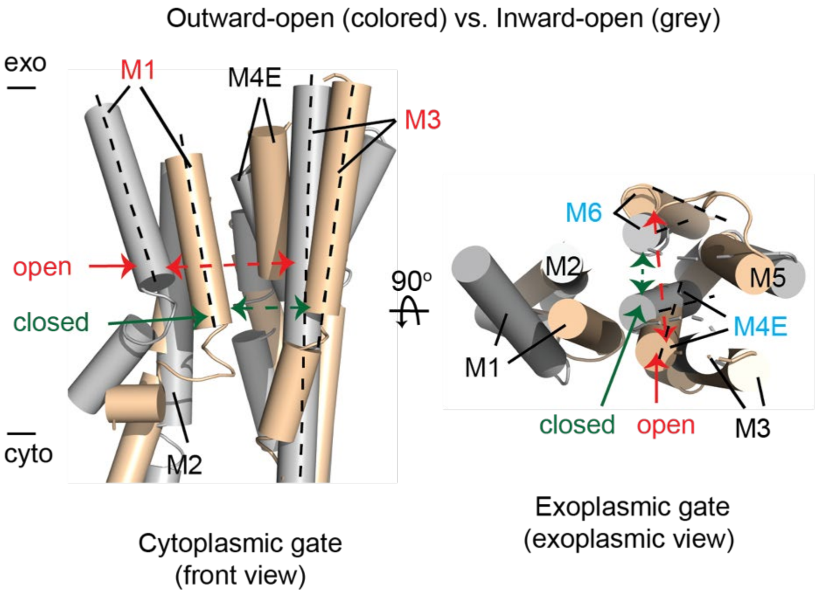

Supplementary Fig. 4: Gating mechanism of the Na^+/K^+-ATPases. Superimpose the M1 – M6 helices in the cytoplasmic side-open (grey) and exoplasmic side-open (colored) state of the human α3 reveals gating mechanism at the cytoplasmic and exoplasmic sides. "Open" (in red) indicates the gate is open. "Closed" (in green) indicates the gate is closed. Front view: FXYD6 was shown in the back of the structural model. Exoplasmic view: viewing the plasma membrane from outside the cell (exoplasm).

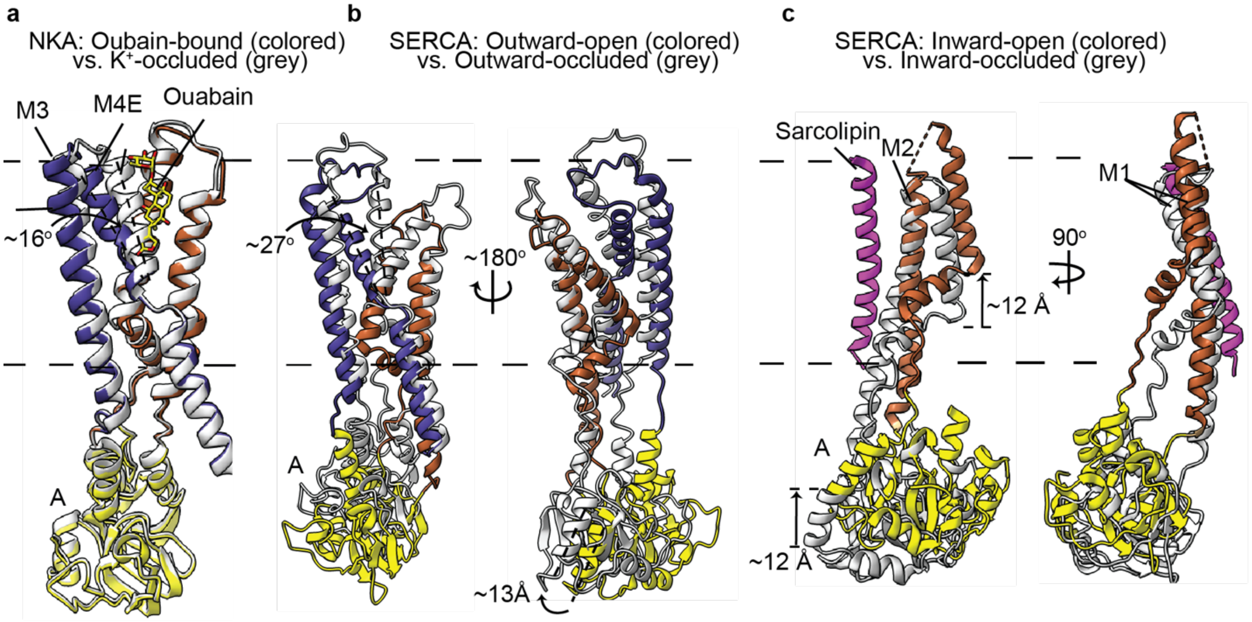

Supplementary Fig. 5: Gating mechanisms of other P-type ATPases. (**a**) Superimpose the ouabain-bound and K^+-occluded structures of the pig Na^+ K^+-ATPase. (**b**) Superimpose the luminal side-open (PDB: 3B9B) and Ca^{2+}-occluded (PDB: 3B9R) structures of the SERCA. (**c**) Superimpose the cytoplasmic side-open (PDB: 4H1W) and Ca^{2+}-occluded (PDB: 1T5T) structures of the SERCA.

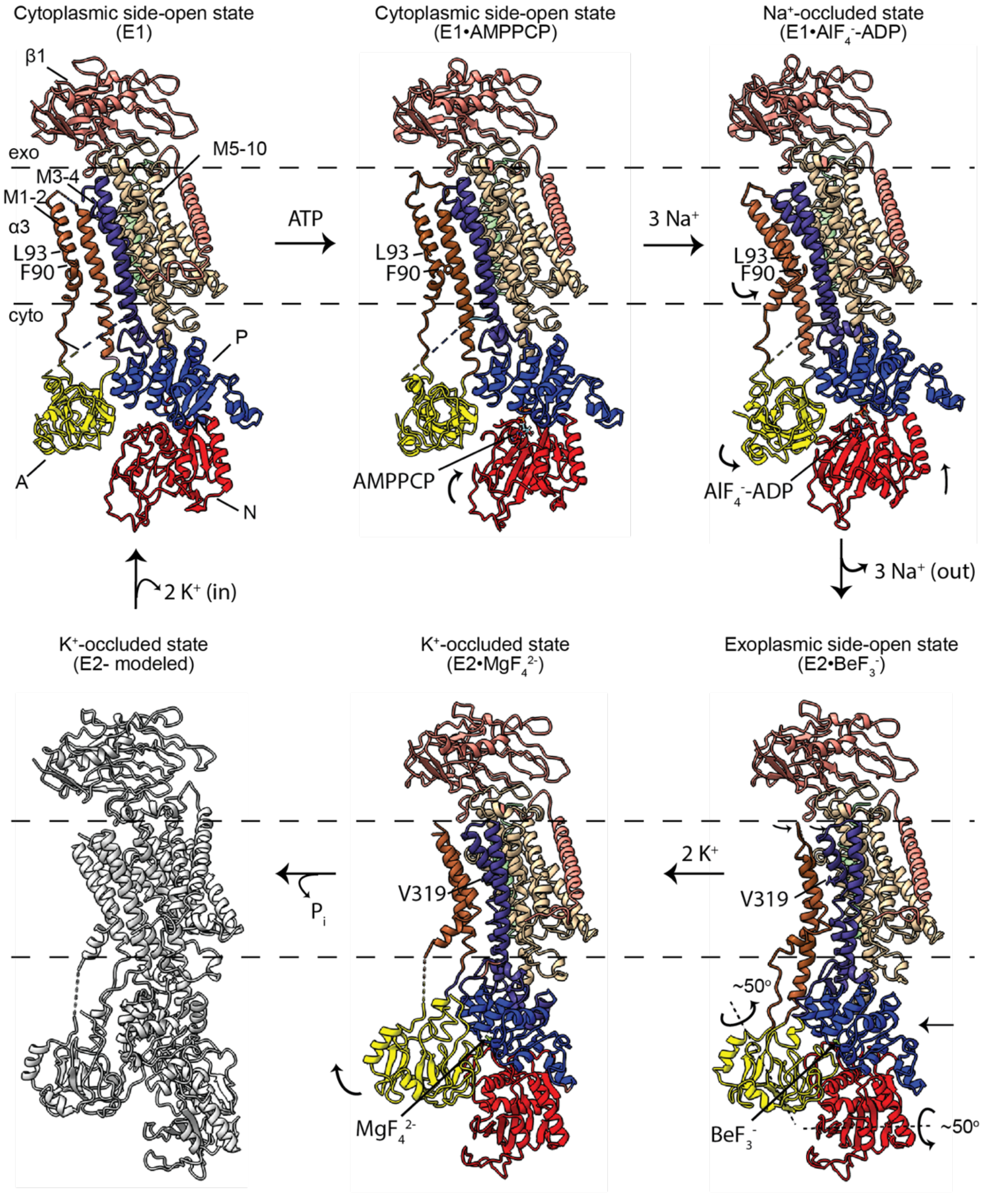

Supplementary Fig. 6: Transport cycle of the human Na^+/K^+-ATPase. A proposed mechanistic model of the human α3 Na^+/K^+-ATPase is shown with five different intermediate structurers

(colored) representing the cytoplasmic side-open (E1), the AMPPCP-bound cytoplasmic side-open (E1•ATP), the Na^+-occluded (E1•P-ADP), the exoplasmic side-open (E2P), and the K^+-occluded (E2•P_i) states and one modeled structure representing the E2 state (grey) based on the Post – Albers scheme. The black dashed lines in the structural models represent missing density of amino acid residues 260 – 271 (cytoplasmic side-open and AMPPCP-bound cytoplasmic side-open), 261 – 270 (Na^+-occluded) and 108 – 114 (exoplasmic side-open).

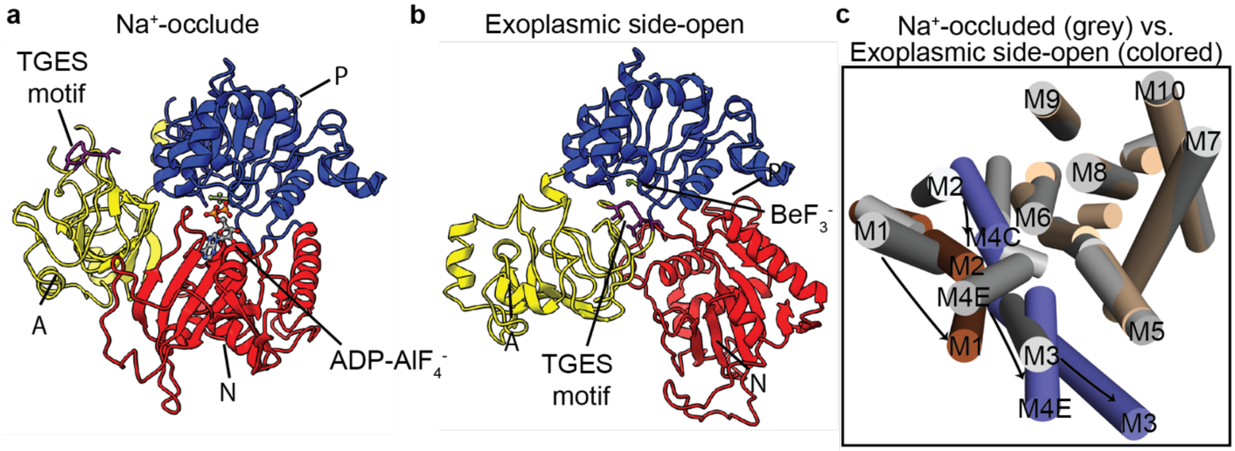

Supplementary Fig. 7: Conformational changes of the E1-to-E2 transition. (**a**) the cytoplasmic domains in the Na^+-occluded (E1•P-ADP) (**a**) and exoplasmic side-open (E2P) **(b)** states. TGES motif (purple) undergoes a large rotation from the E1•P-ADP to E2P state. **(c)** Superimpose the alpha's transmembrane helices of the cytoplasmic side-open (colored) and Na^+-occluded (grey) structures by aligning the M7 - M10 helices (exoplasmic view). Exoplasmic view: viewing the plasma membrane from outside the cell (exoplasm).

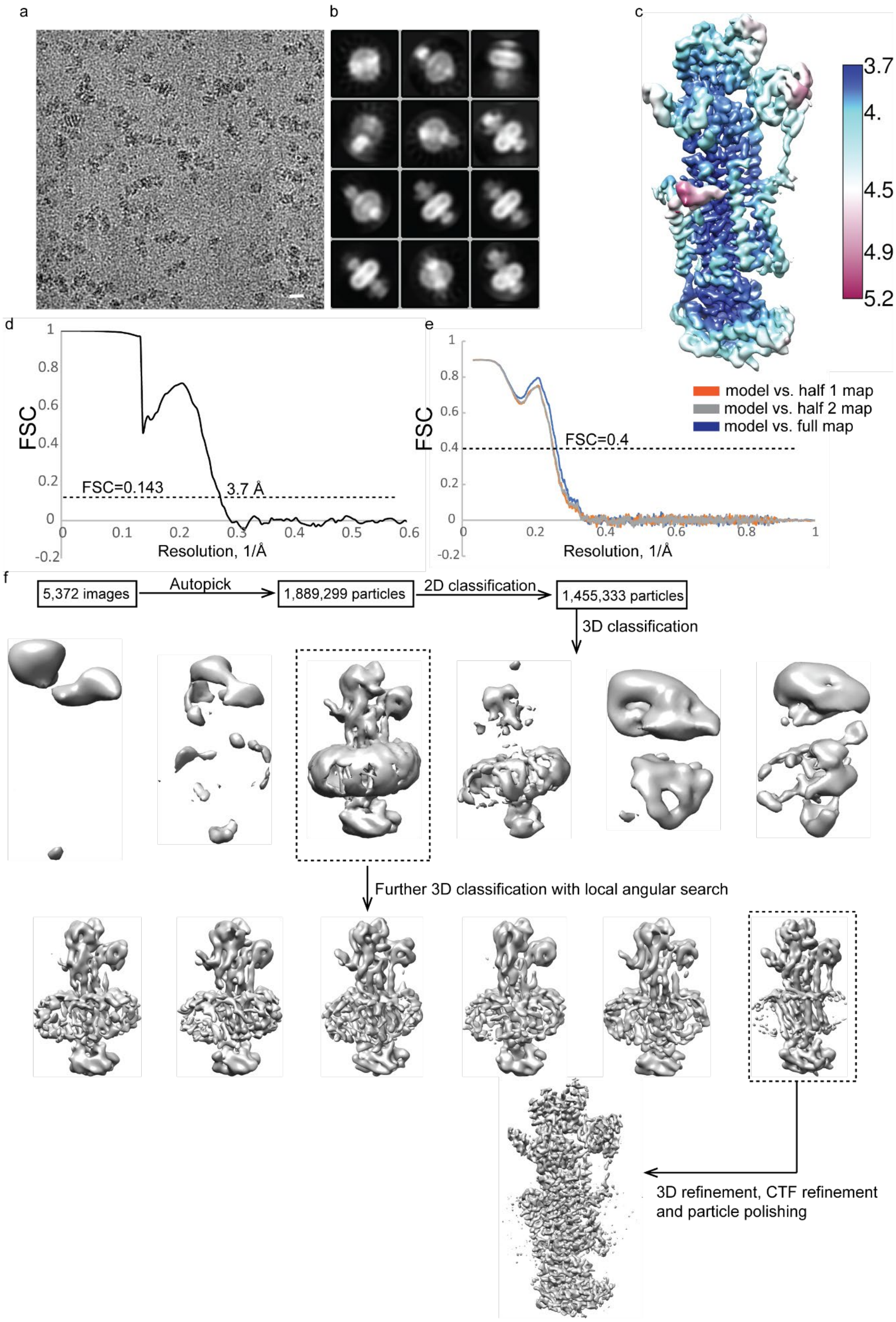

a
b
c
3.7
4.
4.5
4.9
5.2
d
FSC
Resolution, 1/Å
FSC=0.143
3.7 Å
e
model vs. half 1 map
model vs. half 2 map
model vs. full map
FSC=0.4
f
5,372 images
Autopick
1,889,299 particles
2D classification
1,455,333 particles
3D classification
Further 3D classification with local angular search
3D refinement, CTF refinement and particle polishing

Supplementary Fig. 8: Flow chart of cryo-EM image processing for the human α3 Na^+/K^+-ATPase in the Na^+-occluded state. (**a**) Representative micrograph from 5,372 images. Scale bar: 200 Å. (**b**) Representative 2D classes. (**c**) Final reconstruction with colors based on local resolution. (**d**) Gold-standard FSC curve of the final 3D reconstruction. (**e**) FSC curves of model vs. full map (blue trace) and model vs. half maps (orange and grey traces). (**f**) Image processing procedure.

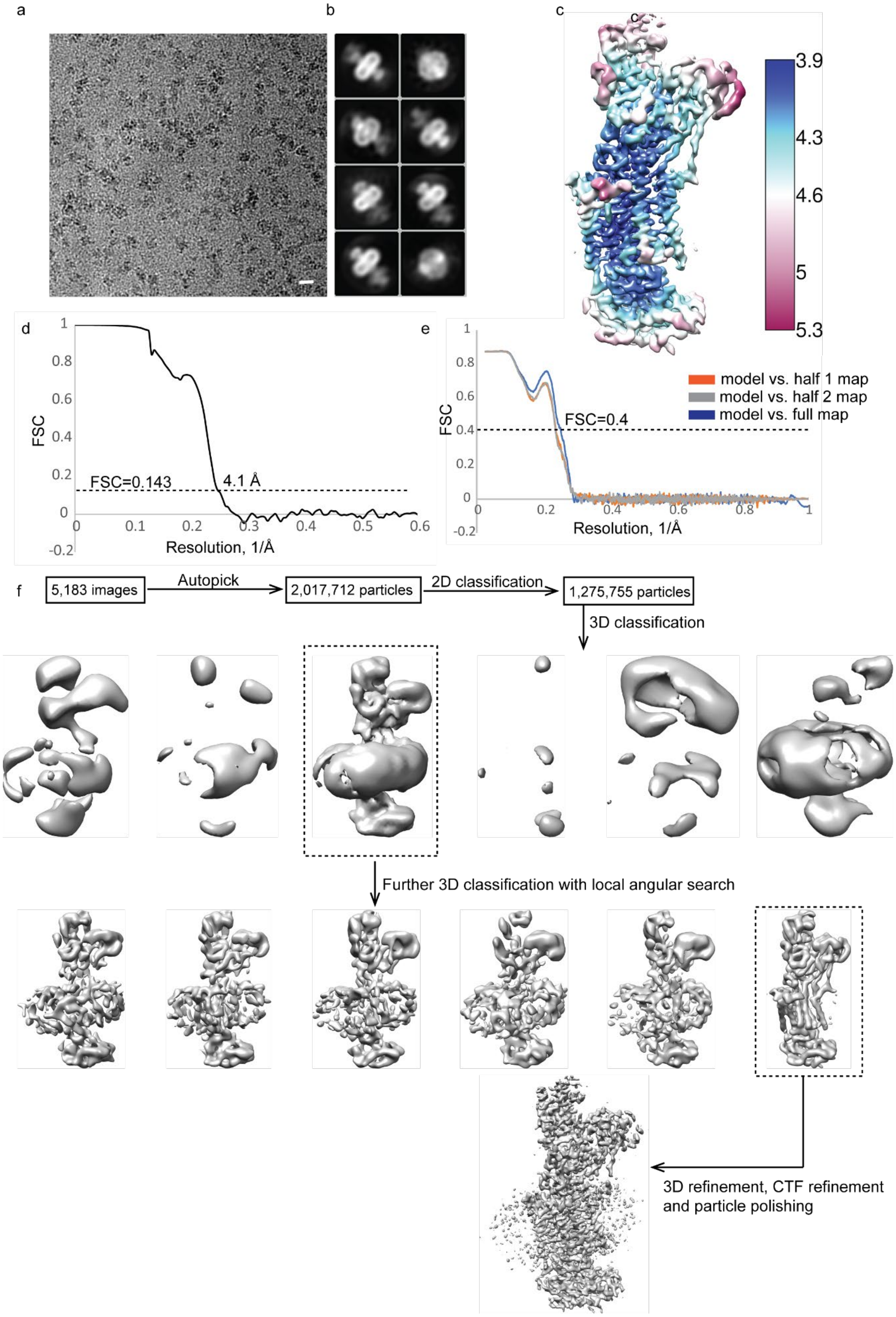

a
b
c
3.9
4.3
4.6
5
5.3
d
FSC
Resolution, 1/Å
FSC=0.143
4.1 Å
e
model vs. half 1 map
model vs. half 2 map
model vs. full map
FSC=0.4
FSC
Resolution, 1/Å
f
5,183 images
Autopick
2,017,712 particles
2D classification
1,275,755 particles
3D classification
Further 3D classification with local angular search
3D refinement, CTF refinement
and particle polishing

Supplementary Fig. 9: Flow chart of cryo-EM image processing for the human α3 Na^{+}/K^{+}-ATPase in the K^{+}-occluded state. (**a**) Representative micrograph from 5,183 images. Scale bar: 200 Å. (**b**) Representative 2D classes. (**c**) Final reconstruction with colors based on local resolution. (**d**) Gold-standard FSC curve of the final 3D reconstruction. (**e**) FSC curves of model vs. full map (blue trace) and model vs. half maps (orange and grey traces). (**f**) Image processing procedure.

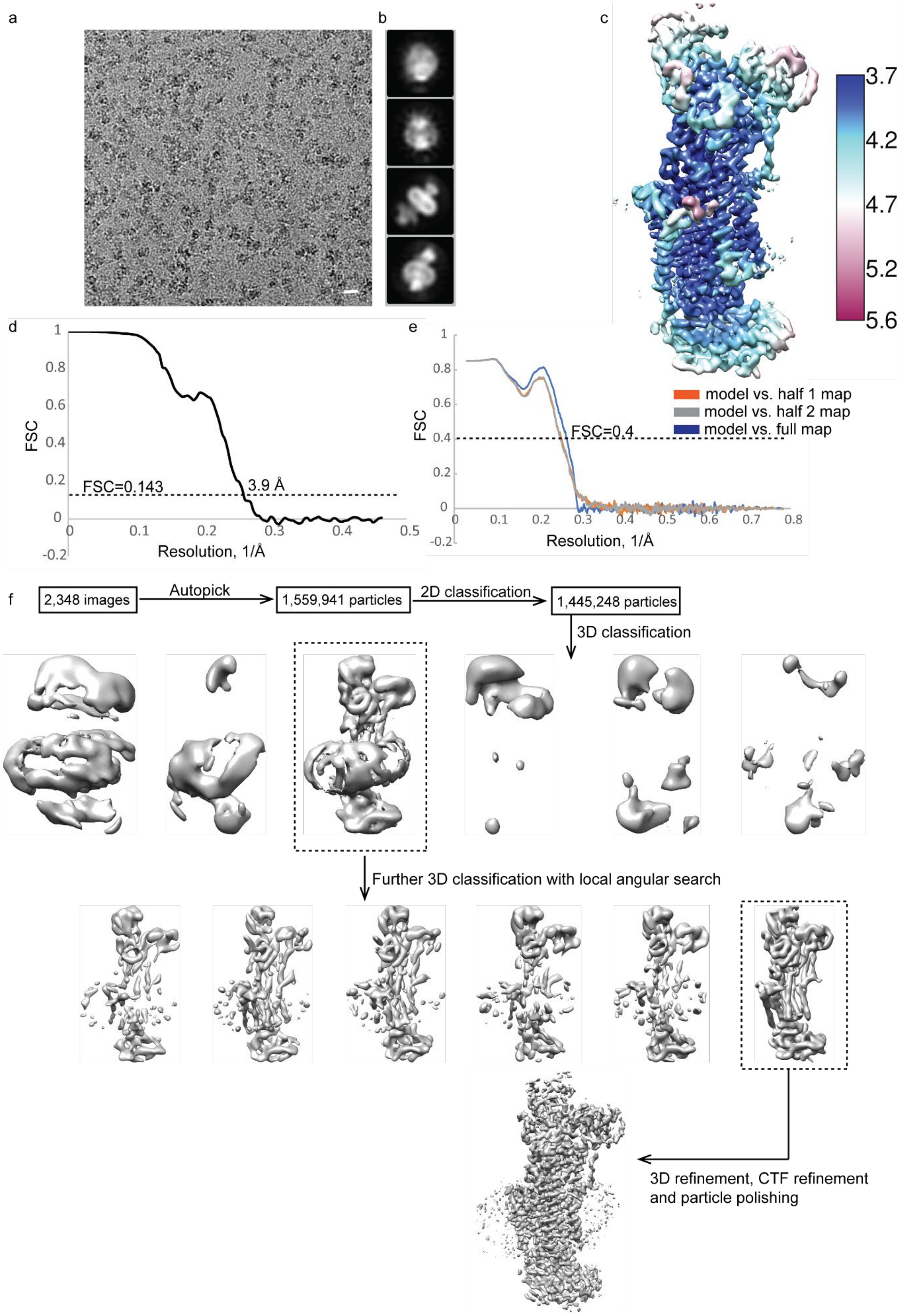
a
b
c
3.7
4.2
4.7
5.2
5.6
d
FSC
Resolution, 1/Å
FSC=0.143
3.9 Å
e
FSC=0.4
model vs. half 1 map
model vs. half 2 map
model vs. full map
f
2,348 images
Autopick
1,559,941 particles
2D classification
1,445,248 particles
3D classification
Further 3D classification with local angular search
3D refinement, CTF refinement
and particle polishing

Supplementary Fig. 10: Flow chart of cryo-EM image processing for the human α3 Na^+/K^+-ATPase in the exoplasmic side-open state. (**a**) Representative micrograph from 2,348 images. Scale bar: 200 Å. (**b**) Representative 2D classes. (**c**) Final reconstruction with colors based on local resolution. (**d**) Gold-standard FSC curve of the final 3D reconstruction. (**e**) FSC curves of model vs. full map (blue trace) and model vs. half maps (orange and grey traces). (**f**) Image processing procedure.

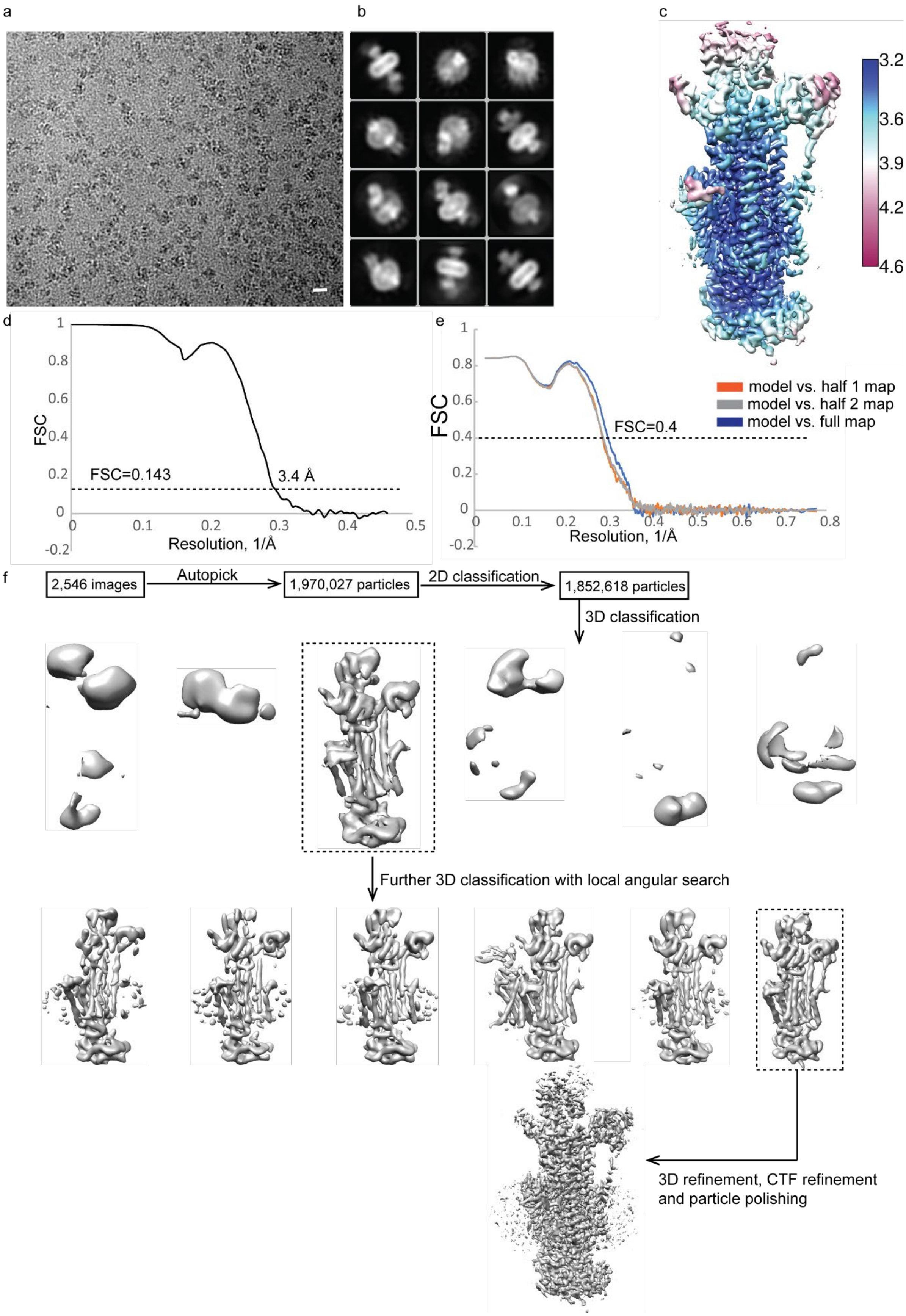
a
b
c
3.2
3.6
3.9
4.2
4.6
d
FSC
FSC=0.143
3.4 Å
Resolution, 1/Å
e
FSC
model vs. half 1 map
model vs. half 2 map
model vs. full map
FSC=0.4
Resolution, 1/Å
f
2,546 images
Autopick
1,970,027 particles
2D classification
1,852,618 particles
3D classification
Further 3D classification with local angular search
3D refinement, CTF refinement and particle polishing

Supplementary Fig. 11: Flow chart of cryo-EM image processing for the human α3 Na^+/K^+-ATPase in the cytoplasmic side-open state. (**a**) Representative micrograph from 2,546 images. Scale bar: 200 Å. (**b**) Representative 2D classes. (**c**) Final reconstruction with colors based on local resolution. (**d**) Gold-standard FSC curve of the final 3D reconstruction. (**e**) FSC curves of model vs. full map (blue trace) and model vs. half maps (orange and grey traces). (**f)** Image processing procedure.

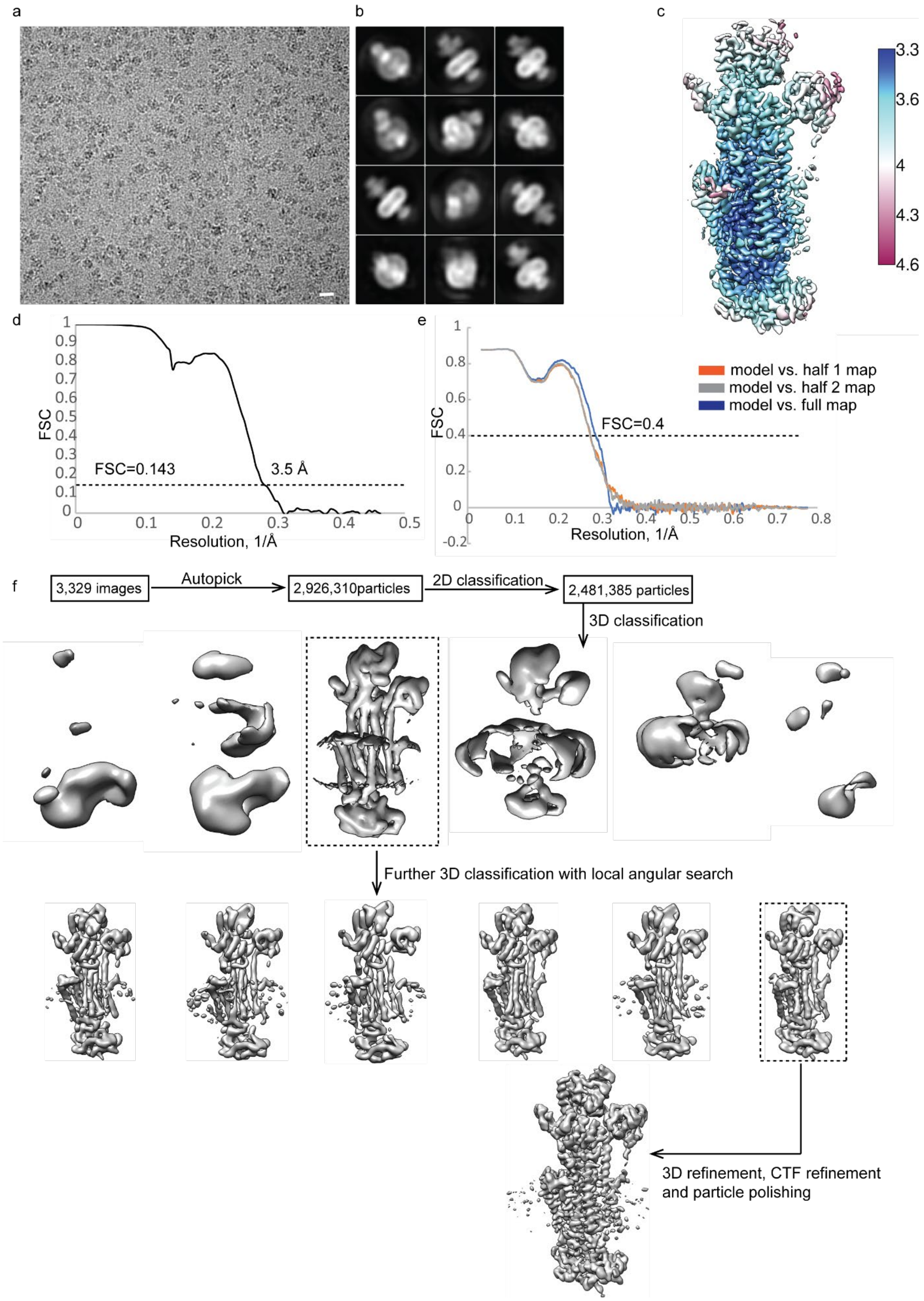
a
b
c
3.3
3.6
4
4.3
4.6
d
FSC=0.143
3.5 Å
FSC
Resolution, 1/Å
e
model vs. half 1 map
model vs. half 2 map
model vs. full map
FSC=0.4
FSC
Resolution, 1/Å
f
3,329 images
Autopick
2,926,310particles
2D classification
2,481,385 particles
3D classification
Further 3D classification with local angular search
3D refinement, CTF refinement
and particle polishing

Supplementary Fig. 12: Flow chart of cryo-EM image processing for the human α3 Na^+/K^+-ATPase in the AMPPCP-bound cytoplasmic side-open state. (**a**) Representative micrograph from 3,329 images. Scale bar: 200 Å. (**b**) Representative 2D classes. (**c**) Final reconstruction with colors based on local resolution. (**d**) Gold-standard FSC curve of the final 3D reconstruction. (**e**) FSC curves of model vs. full map (blue trace) and model vs. half maps (orange and grey traces). (**f)** Image processing procedure.

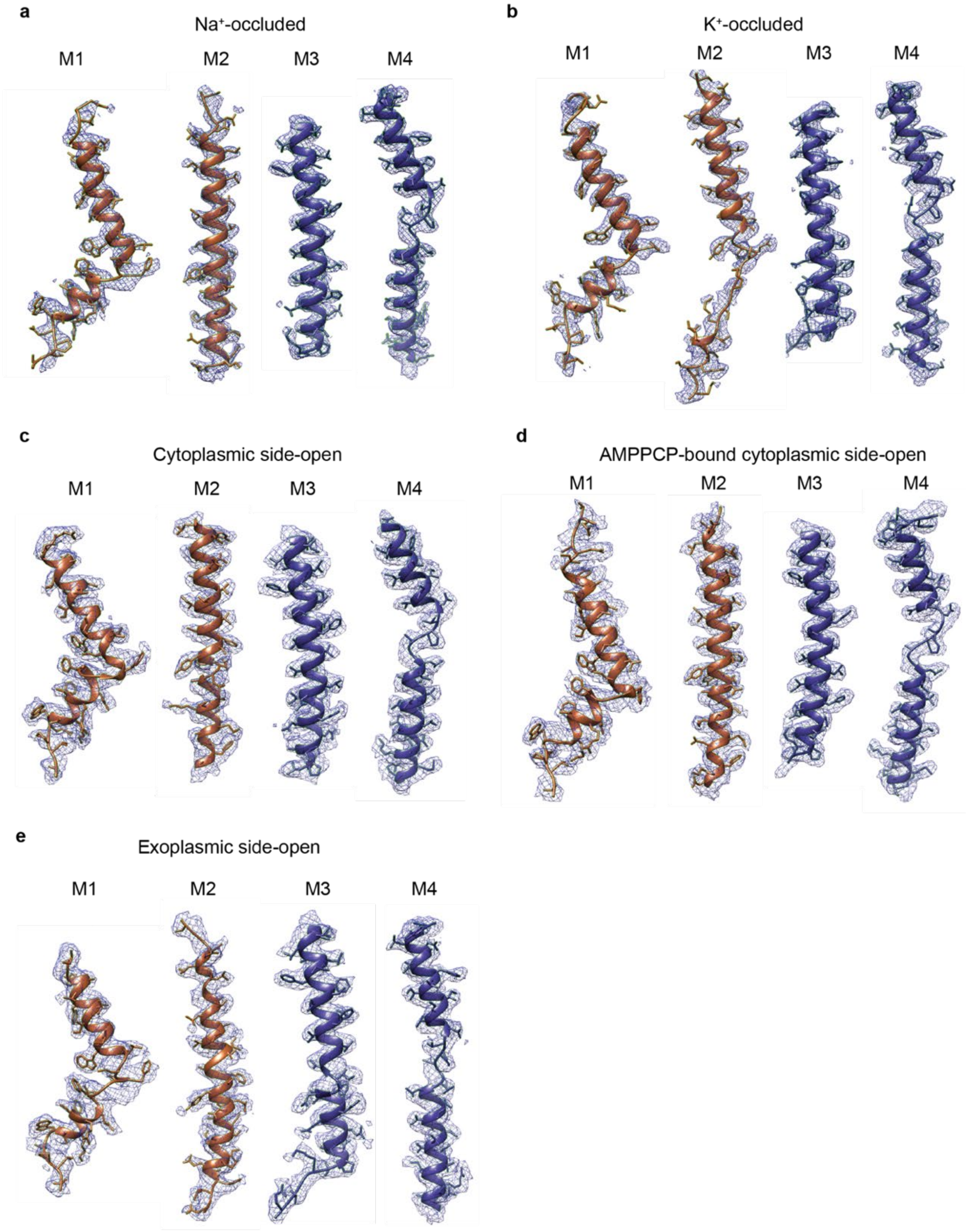

Supplementary Fig. 13: Representative regions of the cryo-EM maps of the Na^+/K^+-ATPase highlight key gating transmembrane helices M1 – M4 in each state. (**a**) Na^+-occluded, (**b**) K^+-

occluded, (**c**) cytoplasmic side-open, (**d**) AMPPCP-bound cytoplasmic side-open, (**e**) exoplasmic side-open.
